# Pure Bourbon Whisky

**Published:** 1868-07

**Authors:** 


PURE BOURBON WHISKY.—It will be seen by the readers of our advertisement sheet that our market is now supplied with a pure and reliable stimulant in the article of whisky from William T. Cutter, who was induced to furnish the same in New York by request of several of the leading physicians who have set their seal of approbation upon it. We have received samples from the agent, William King, jr., and can assure the profession that it is a very pleasant, and we have no doubt a very pure and reliable article, well suited for medicinal purposes.

The two sons of Dr. R. Ogden Doremus, of New York, the well-known chemist, while playing in a wooden play-house at the back of their residence, on the 26th ult., accidentally set it on fire. They were unable to escape immediately, and the younger of the boys perished in the flames.

